# Evolution of mechanical properties of organic-rich shale during thermal maturation

**DOI:** 10.1038/s41598-024-75035-7

**Published:** 2024-10-17

**Authors:** Jianfeng Wang, Dayong Liu, Jianfei Shi, Chao Yang, Yuke Liu, Guozhi Wang, Huijuan Guo, Peng Liu, Yongqiang Xiong, Ping’an Peng

**Affiliations:** 1 State Key Laboratory of Organic Geochemistry, Guangzhou Institute of Geochemistry, Chinese Academy of Sciences, Guangzhou, 510640 China; 2 CAS Center for Excellence in Deep Earth Science, Guangzhou, 510640 China; 3 Key Laboratory of Petroleum Resources Research, Lanzhou, Gansu Province 730000 China; 4 Research Center for Oil and Gas Resources, Northwest Institute of Eco-Environment and Resources, Chinese Academy of Sciences, Lanzhou, 730000 China; 5 Key Laboratory of Natural Gas Hydrate, Guangzhou Institute of Energy Conversion, Chinese Academy of Sciences, Guangzhou, 510640 China; 6 Research Institute of Petroleum Exploration and Development, Beijing, 100083 China; 7 College of Safety Science and Engineering, Xi’an University of Science and Technology, Xi’an, Shanxi 710054 China

**Keywords:** Organic matter, Clay matrix, Organic-rich shale, Thermal maturation, Mechanical properties, Geochemistry, Geology, Mineralogy

## Abstract

Accurate assessment of the mechanical properties of organic matter, clay matrix, and bulk shale during maturation remains a challenge. Here, we aim to assess the mechanical properties of organic-rich shale during maturation using a combination of nanoindentation methods and various geochemical analyses, i.e., mineral composition, mass loss rate, chemical structure of organic matter, and Rock-Eval analyses. Results show that the evolution of mechanical properties of organic matter in shale during maturation can be divided into: the main oil-generation stage, and the condensate oil and gas generation stage. The stiffening of organic matter in the shale is mainly due to increased aromaticity and condensation of aromatic groups. The clay matrix experiences a slight decrease in hardness and Young’s modulus at low maturity levels due to the generation of liquid hydrocarbons. However, overall, the clay matrix becomes stiffer as the shale matures due to shale dehydration, expulsion or cracking of liquid hydrocarbons, transformation of clay minerals, and hardening of organic matter. The Young’s modulus and hardness of bulk shale generally increase with increasing maturity. This is closely related to the hardening of organic matter and clay matrix, as well as the development of the more compact and dense microstructure in the shale.

**Supplementary Information:**

The online version contains supplementary material available at 10.1038/s41598-024-75035-7.

## Introduction

**Figure 1:**
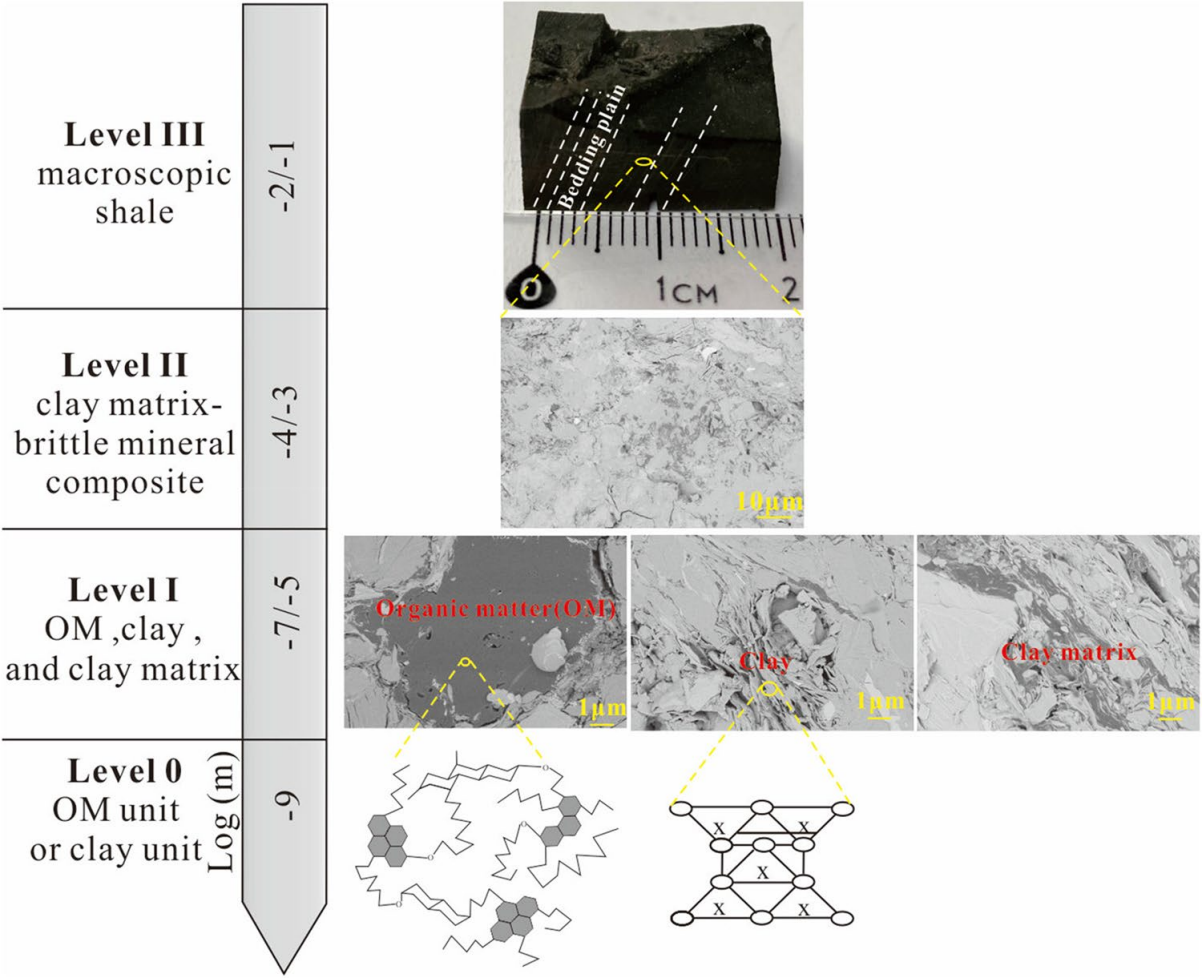
Multi-scale structure for organic-rich shale. From top to bottom, Level III refers to the macroscopic shale sample, and the bedding plain is visible. Level II refers to the scale of clay matrix intermixed with brittle minerals such as silt size quartz or feldspar grains; Level I refers to OM, porous clay minerals and clay matrix in shale under the backscatter electron imaging (BSE); Level 0 refers to unit of OM or clay minerals.

Shale is a common fine-grained sedimentary rock in various field operations, including the extraction of oil and natural gas^1^, the geological storage of radioactive waste^2^, and the storage of carbon dioxide^3,4^. This rock is particularly inhomogeneous, containing a mix of inorganic minerals (e.g., quartz, feldspar, carbonate and pyrite) and a certain amount of organic matter (OM) and defects (pores and fractures)^5–11^. For example, the organic-rich shales from the Upper Ordovician to lower Silurian Wufeng and Longmaxi formation successions in the Sichuan Basin, China, have the total organic matter (TOC) content of typically ≥ 2 wt.%^12^. The mechanical behavior of organic-rich shale is related to its composition and structure, which can be divided into four levels from the nanoscale to the macroscopic scale (see multi-scale structure in Fig. 1). OM in shale can not only produce oil/gas^13^, but also affect the macroscopic mechanical behavior of shale^14,15^, especially for organic-rich shales (e.g., TOC content ≥ 10%). For example, when the TOC content of the Bakken Formation shale increases from 7 wt% to 20 wt%, its calculated Young’s modulus decreases from 45.7 GPa to 20.1 GPa^14^. Besides, shale is formed by compaction, dehydration and cementation of clay minerals. The fine-grained platy clay mineral particles can form clay matrix with other relatively small sizes of minerals, such as carbonate minerals, pyrite, and OM^16–19^. For the clay-rich shale, clay matrix can greatly influence the mechanical properties of the bulk shale. Additionally, as the maturity increases, the OM will produce the hydrocarbons and its own chemical structure will change^20,21^, thus its mechanical properties will vary in the process of thermal evolution^22–25^. On the other hand, different types of water, i.e., free water, adsorbed water, and crystal water in the shale, can escape during the process of thermal evolution, which will change the mechanical properties of shale matrix, and further influence the mechanical properties of bulk shale^26^. These changes may be the main influencing factors leading to uncertainty in the macroscopic mechanical properties of organic-rich shale. Therefore, the comprehensive investigation of the changes in mechanical properties of OM, clay matrix, and the bulk shale with maturity at the microscopic level is crucial for developing rock mechanics physical models, and will greatly help us optimize hydraulic fracturing design and evaluate drilling stability^25,27–30^.

Conventional uniaxial and triaxial compression tests could characterize mechanical properties of shale at the macroscale^31,32^. However, they have some intrinsic limitations, including the demand of standard inch-sized samples, low repeatability of experiments, relatively high cost and long experiment period. Nanoindentation can overcome these drawbacks and has gradually become an alternative technique for obtaining the mechanical properties of shale^33–35^. This technology only requires a small amount (several centimeters in size) of rock debris samples, and can quickly acquire mechanical properties of shale components and the bulk shale by achieving the depth of penetration into the sample surface from several tens of nanometers to several micrometers^36^. In the past few years, the nanoindentation technique has been utilized to analyze mechanical properties of various individual phases in shale, such as OM^15,37–42^ and individual minerals^7,43–48^, and different composite phases in shales based on the deconvolution results^14,49,50^, as well as bulk mechanical parameters^29,38,51,52^ and the elastic anisotropy of shale^44,53^. Therefore, it is possible to obtain the variations of mechanical properties of bulk shale or its components (e.g., OM, clay matrix) with maturity from the microscale using nanoindentation.

OM (e.g., kerogen) is a geological polymer with diverse macromolecular compositions, exhibiting a wide range of molecular weights and compositions^13,22^. During the geological evolution process of deep burial, its structure and chemical components vary greatly with increasing maturity^22,54–59^. This change may lead to the formation of different microstructures and the alteration of initial mechanical properties^60^. As a result, it is vital to characterize how the structural changes of OM during the maturation process affect the evolution of its mechanical properties. So far, solid-state nuclear magnetic resonance (NMR) spectroscopy has been used to characterize the chemical structural changes of OM during maturation^54,61–64^. It could provide accurate quantitative results on the distribution of different types of functional groups in OM, such as, various structural parameters (i.e., the proportion of aliphatic carbon and aromatic carbon). During the maturation process of OM, the proportion of aliphatic carbon (f_al_) decreases, while the proportion of aromatic carbon (f_ar_) increases, and the contraction between aromatic sheets leads to structural condensation of OM^20,21,62,64–67^, which may further change the mechanical properties of OM. Previous studies have investigated the evolution of mechanical properties of pure kerogen and solid bitumen extracted from the shale rock by the thermal simulation experiment, suggesting that their mechanical parameters of them increase with increasing maturity^30,68^. On the other hand, the mechanical properties of clay matrix^14,49^ and bulk shale^69,70^ can also vary with maturity. However, to the best of our knowledge, no attempt was made to obtain the variation of mechanical properties during the whole thermal evolution stage (from low- to over-maturity) for OM and clay matrix in shale.

In this study, the original immature organic-rich shale from the Yanchang Formation, Ordos Basin, China was used to prepare a series of shale samples with different maturities by the thermal simulation experiment. Then, the artificially prepared shale samples at different maturities were measured by^13^C NMR to determine the variation of chemical structures for OM. Meanwhile, the shale samples were analyzed by X-ray diffraction (XRD) for mineralogy, Rock-Eval tests for geochemical parameters, total organic content (TOC), and nanoindentation test for mechanical properties. The main objectives of this study are: (1) to locate OM in shale and examine the main factors influencing the Young’s modulus of OM in shale; (2) to analyze the changes in mechanical properties of the clay matrix in shale during thermal maturation; and (3) to evaluate the evolution of mechanical properties in bulk shale with increasing maturity and discuss the primary controlling factors.

## Materials and methods

### Shale samples

The original shale has a high TOC (12.01 wt%) and an OM type of II_1_, and a low vitrinite reflectance (*R*_o_) value of 0.55%. It is at an immature stage and is very suitable for simulation experimental research. We cut the rock core with a thickness of about 5 cm along the vertical bedding direction into multiple cylindrical samples, and then grind them into near-cylindrical shapes to conduct thermal simulation experiments at different temperature points.

### Rock-Eval and pyrolysis experiment

Rock-Eval 6 (Vinci Technologies, France) was used to obtain the geochemical parameters of the shale samples. We grinded the shale samples to a particle size of ~ 100 mesh, and approximately 60–70 mg of the shale sample at temperature point was heated from room temperature to 300 ^o^C under nitrogen, then increased from 300 ^o^C to 650 ^o^C at a rate of 25 ^o^C/min. Pyrolysis experiment was performed to simulate the process of oil generation from shale samples of different maturities^71,72^ and the variation of mechanical properties of bulk shale^69,70^ and OM^30,68^. The column shale sample was placed in a high-pressure vessel and buried with 60 mesh quartz sand. A vertical pressure of 40 MPa through a jack was set. We performed the artificial maturation on the column shale to obtain conditions as close as possible to the natural conditions of sedimentary basins^73–75^. The high-pressure vessel was heated to 250 ^o^C in about half an hour, then raised to the specified temperature point at a rate of 1 ^o^C/min and kept at a constant temperature for 24 h. We set 9 temperature points in the temperature range of 320–500 ℃, each temperature point corresponded to a sample maturity, and the maturity was denoted using Easy*R*_o_^76^. Nine shale samples were collected for each temperature point: 320 ℃ (Easy*R*_o_ 0.66%), 340 ℃ (Easy*R*_o_ 0.75%), 360 ℃ (Easy*R*_o_ 0.87%), 380 ℃ (Easy*R*_o_ 1.04%), 400 ℃ (Easy*R*_o_ 1.26%), 420 ℃ (Easy*R*_o_ 1.52%), 440 ℃ (Easy*R*_o_ 1.82%), 460 ℃ (Easy*R*_o_ 2.16%), and 500 ℃ (Easy*R*_o_ 2.91%).

### X-ray diffraction (XRD)

X-ray diffraction analysis was performed by a Rigaku MiniFlex-600 X-ray diffractometer with a Cu tube (monochromatic). The operating voltage and current were set to 40 kV and 30 mA, respectively. The slit width was 1 mm and the scanning range was 2θ = 3 ~ 80º with the scanning speed of 10 º/min. We calculated the mineral content by correcting the peak area of different minerals using the Lorentz polarization method.

### Solid-state13C nuclear magnetic resonance (NMR)

Solid-state^13^C NMR was used to obtain carbon structure information of OM in the sample. A Bruker AVANCE III 400 MHz with a 4-mm probe, a spectral width of 100 kHz, and a spinning speed of 14 kHz was used to proceed this analysis^77^. The detection resonance frequency of^13^C, sampling time, and cycle delay time were 100.613 MHz, 5.12 µs and 1 s, respectively. The DP/MAS (Direct Polarization/magic-angle spinning) solid-state^13^C NMR method was applied^62^.

### Nanoindentation measurements

**Figure 2:**
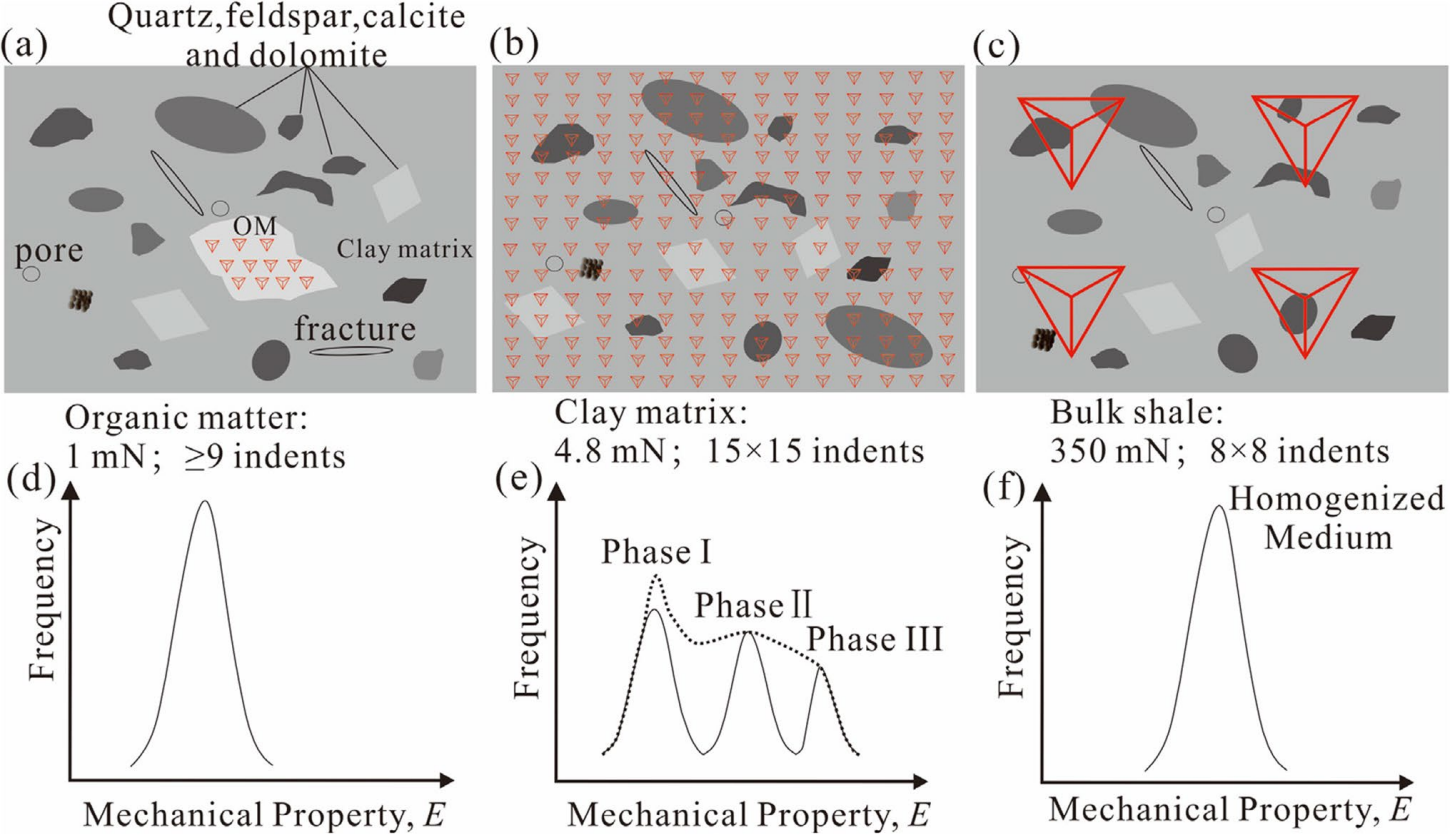
The schematic diagram for calculating the Young’s modulus (E) of organic matter (OM) (**a**, **d**), clay matrix (**b**, **e**), and the bulk shale (**c**, **f**). (**a**) refers to indents produced by a peak load of 1mN and located solely on the OM. (**b**) refers to indents produced by a peak load of 4.8mN, e.g., a 15 × 15 indentation pattern, located on different minerals. These indents can yield Young’s modulus values for three different phases. We assume Phase I represents the clay matrix (**e**). (**c**) refers to indents created by a peak load of 350mN, with an 8 × 8 indent pattern, located on multiple phases of shale. These indents produce a Young’s modulus value for a homogeneous medium (**f**), and we assume that this mean value can represent the Young’s modulus of the bulk shale.

We conducted the nanoindentation tests using Anton Paar TTX NHT^3^ nanoindenter, equipped with a diamond Berkovich tip with a radius of curvature of 50–100 nm. The indentation system has a high-resolution optical microscope, making it possible to observe the surface and locate the OM clearly before the nanoindentation tests were conducted^7,48^. The grid nanoindentation experiment was performed on the OM, clay matrix and the bulk shale (Fig. 2a and c). We selected a suitable matrix based on the size of the OM using the peak load of 1mN with the distances of 10 μm between neighbouring indentations (Fig. 2a) and the mean value can represent the Young’s modulus of the OM (Fig. 2d). The 13 × 13 or 15 × 15 indents using the peak load of 4.8 mN with the distances of 20 μm between neighbouring indentations to obtain the Young’s modulus of three different mineral phases^14^, we assume Phase I is the clay matrix (Fig. 2b and e). The peak load was 350 mN and a nanoindentation grid of 8 × 8 (64 indents in total) was applied to cover an area of ~ 700 μm × 700 μm to obtain the mean Young’s modulus value of bulk shale (Fig. 2c and f). The nanoindentation test contains loading stage (30s), holding stage (2s) and unloading stage (30s). Hardness (*H*), Young’s modulus (*E*), the maximum indentation depth (*h*_max_), and *W*e (the elastic work)/total work (*W*t) are determined based on the formula based on previous studies^36,78^.

### Scanning electron microscopy-energy dispersive spectroscopy (SEM-EDS)

After nanoindentation tests, a Hitachi S8010 SEM system equipped with secondary electron, backscattered electron detectors, and energy dispersive spectroscopy was utilized to observe the indents from 350 mN at the surface of the rock. The accelerating voltage is 1.5 kv and working distance is 3–8 mm. We imaged using SEM-EDS, and analyzed the size of the indents and identified the main minerals based on the morphological characteristics and EDS element intensities.

## Results

### Organic petrography

The shale displays an abundance of organic macerals, including liptinite, vitrinite, inertinite, and sapropelinit^10^. Notably, sapropelinite displays a widespread occurrence within this shale. Here, the macerals show in the Yanchang shale were identified by analysing the characteristics of OM under reflected light and fluorescence using an optical microscope. Under oil-immersion reflected light, vitrinite appears as dark gray to gray, exhibiting flat protrusions, yet it lacks fluorescence (Fig. S1a). Conversely, inertinite exhibits a bright white colour under oil immersion reflection, without protrusions and fluorescence (Fig. S1a). Sporinite, a subtype of liptinite, often displays fluorescence colors ranging from greenish-yellow to orange-yellow, distributed in sharp-edged, short-columnar forms (Fig. S1b). Alginite, which belongs to the sapropelinite group, is predominantly oval in shape and has a strong, bright yellow fluorescence (Fig. S1c).

### Mass loss rate, Rock-Eval data, and mineral content of bulk shale

**Figure 3:**
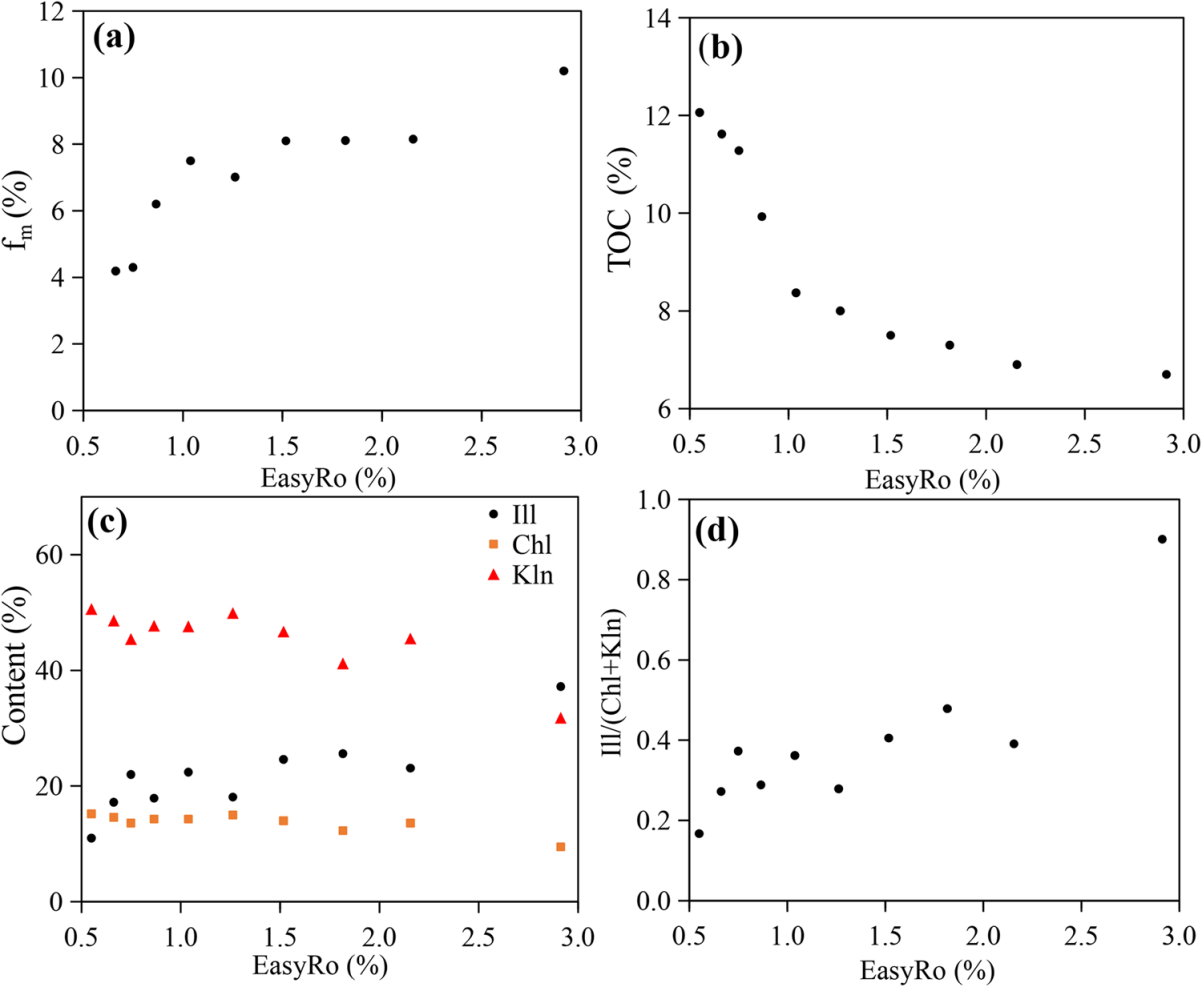
Plots of (**a**) mass loss rate (f_m_), (**b**) TOC content, (**c**) the fraction of various clay minerals, and (d) the ratio of illite to (chlorite + kaolinite). Kaolinite=Kln, Chlorite=Chl, and Illite=Ill.

The original shale block samples were weighed before and after the simulation experiment, thus, the mass loss rate of shale (f_m_) could be obtained after the simulation experiment. As shown in the Fig. 3a, the higher value the Easy*R*_o_ was, the greater the mass loss of shale was measured. The evolutionary trend can be divided into two stages. Stage 1: the original sample is heated from 25 ℃ to 380 ℃ (Easy*R*_o_=0.55%~1.04%). The mass loss of shale after simulated heating increases rapidly with the increase of experimental temperature. Stage 2: Easy*R*_o_=1.04 ~ 2.91%. the maturity continues to increase, while the increase in mass loss rate slows down. Figure 3b shows that the TOC of shale decreases with increasing maturity. Specifically, it slightly decreases from 12.06% at *R*_o_=0.55–11.28% at EasyRo = 0.75%, then decreases markedly to 8.37% at Easy*R*_o_=1.04%, before decreasing further at a lower rate (Fig. 3a; Table S1). We also obtained the residual organic carbon (f_RT_):the residual TOC of shale/the original TOC of shale×(1-f_m_), which represented the loss of OM abundance (Table S1). The variation of the f_RT_ value with maturity is similar to that of TOC value. The results from Rock-Eval are shown in Table S1. Before Easy*R*_o_=1.04%, the S_1_ and S_2_ decrease rapidly, suggesting that a large amount of OM is cracked to produce hydrocarbons. The T_max_ (temperature for maximum generation rate of hydrocarbons) increases with the increase of experimental temperature. The variation of HI (S_2_/TOC×100) value is similar to that of S_2_ value (Table S1).

The relative content of hard minerals (quartz, feldspar, etc.) and clay minerals (kaolinite, illite, chlorite) in all shale samples is basically consistent. However, as the maturity increases, there are significant differences in the content of different types of clay minerals (Fig. 3c; Table S2). The relationship of clay minerals content for shale samples is: kaolinite > illite > chlorite. As maturity increases, the content of kaolinite and chlorite decreases, especially for kaolinite, while the content of illite increases (Fig. 3c). The ratio of illite to (chlorite + kaolinite) content also shows an increasing trend with the increase of maturity (Fig. 3d). This indicates that during the thermal maturation process of shale, chlorite and kaolinite are transformed into illite.

### Chemical structure of organic matter

**Figure 4:**
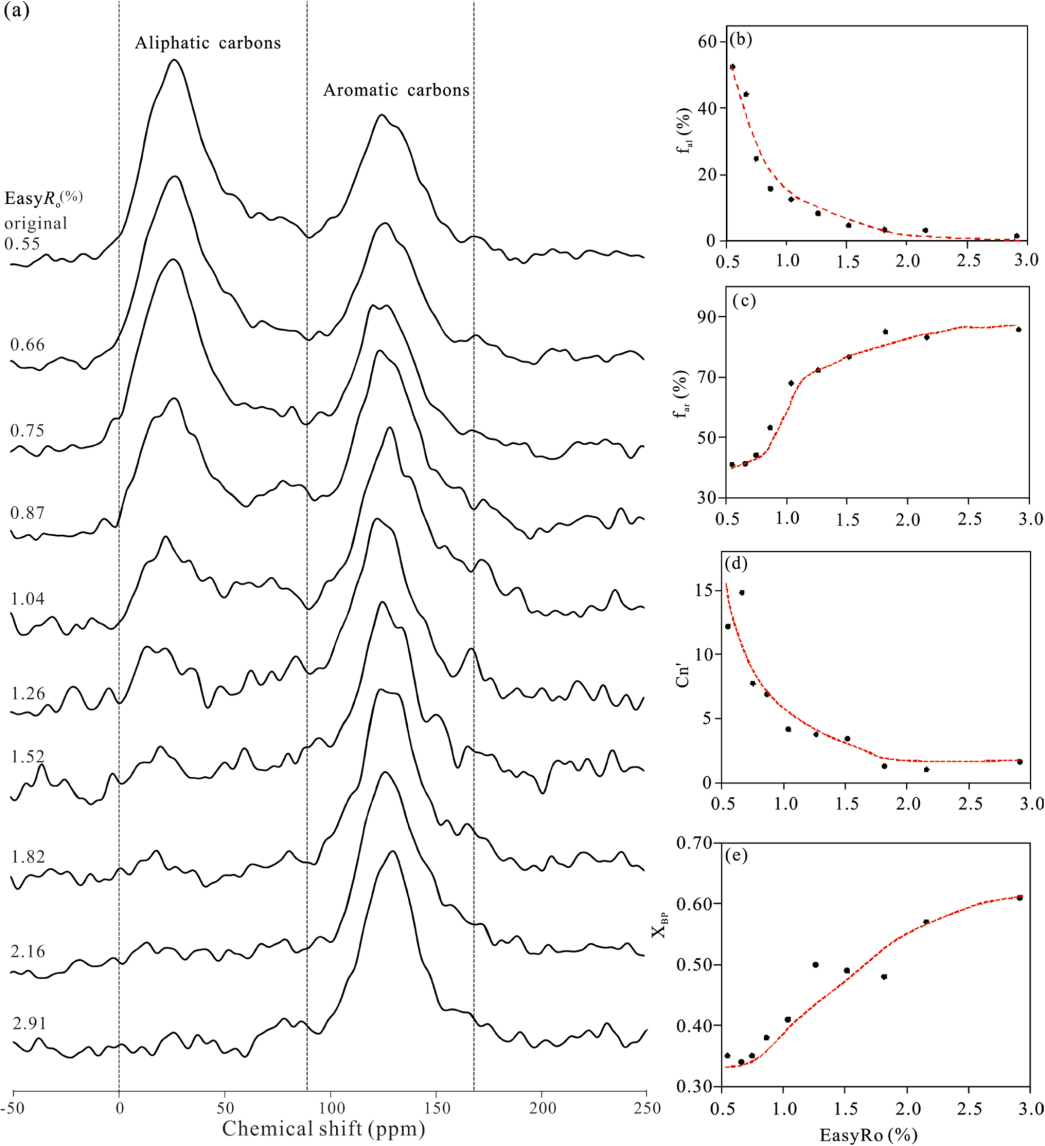
Solid state^13^C CP-TOSS/MAS NMR spectra in order of increasing maturity (**a**), the fraction of aliphatic carbon (f_al_) (**b**), aromatic carbon (f_ar_) (**c**), aliphatic chain length Cn’ (**d**) and ratio of bridgehead carbon to aromatic peripheral carbon (X_BP_) (**e**) of OM versus Easy*R*_o_%.

At the immature to low maturity stages (Easy*R*_o_ < 1.04%), two prominent peaks are observed, representing aliphatic carbon with chemical shifts ranging from 5 to 95 ppm and aromatic carbon with shifts ranging from 95 to 150 ppm (Fig. 4a)^62,64,67^. The aliphatic carbon proportion (f_al_) exceeds that of aromatic carbon in the original sample, indicating the aliphatic carbon’s dominance in the organic macromolecular structure. However, with increasing thermal maturity, f_al_ gradually decreases, whereas the proportion of aromatic carbon (f_ar_) steadily rises. For samples with Easy*R*_o_ ≥ 1.52%, the^13^C NMR spectrum of shale residuals shows almost a single peak, exclusively representing aromatic carbon.

The f_al_ and f_ar_ evolve reciprocally, with f_al_ gradually decreasing and f_ar_ gradually increasing as maturity increases (Fig. 4b and c). The evolution of the f_ar_ can be broadly categorised into two stages. Stage 1: before Easy*R*_o_=1.04%, the f_ar_ rapidly increases, from 41.1 to 68.0%; stage 2: at Easy*R*_o_≥1.04%, the f_ar_ slowly increases until it remains relatively stable at Easy*R*_o_≥1.52%. Conversely, the f_al_ exhibits a rapid decrease at Easy*R*_o_=0.55–1.04%, followed by a nearly constant level at Easy*R*_o_≥1.04%. More specifically, the decreasing trend of f_al_ closely mirrors the decrease in TOC content observed in Fig. 3b, suggesting that the decrease in OM content during maturation is mainly attributed to the cracking of aliphatic carbon.

Based on the peak fitting for the^13^C NMR spectra of shale residues, aliphatic carbon includes six types of functional groups with aliphatic methyl (f_al_^CH3^), aromatic methyl (f_ar_^CH3^), methylene (f_CH2_), methine (f_CH_), quaternary (f_C_), and O-alkyl (f_O_) carbons, which had chemical shifts of 10–19, 19–22, 22–35, 35–40, 40–55, and 55–95 ppm, respectively^79,80^. Methyl, methylene, and methine are the major aliphatic carbon components of these samples (Table S3). Aromatic carbon includes four major functional groups with protonated (f_a_^H^), bridgehead (f_a_^B^), branched (f_a_^S^), and oxy-aromatic (f_a_^O^) carbons, exhibiting chemical shifts ranging from 95–118, 118–135, 135–145, and 145–155 ppm, respectively^79,80^. The f_a_^H^ and f_a_^B^ values represent the largest aromatic group for all these samples in almost all thermal maturation processes. The f_a_^B^ increases from 10.0 to 30.6% during maturation (Table S3), with an increasing trend similar to that of the f_ar_. The average length of aliphatic chains, the ratio of branched to aliphatic carbon (Cn’), represents the half length of the middle chains or the average length of the side chains connecting aromatic clusters^81,82^. The Cn’ shows a decreasing trend during maturation process (Fig. 4d). The decreasing rate becomes slower with increasing maturity, and almost does not decrease at Easy*R*_o_ > 1.52%, suggesting that there are many branched carbons or few aliphatic carbons in the samples^81^. The ratio of bridgehead aromatic carbon to peripheral carbon, X_BP_, suggests the degree of condensation of aromatic carbon^83^. Its value increases from 0.35 for the original sample to 0.61 at Easy*R*_o_=2.91% (Fig. 4e; Table S3). This means, the decomposition of aliphatic chains and condensation of aromatic groups leads to more polymerization of aromatic rings and presents a multi-ring structure as maturity increases^83^.

### Mechanical properties of organic matter, clay matrix and bulk shale

#### Mechanical properties of organic matter

The optical images of OM in the shale after nanoindentation experiments (Fig. S2) show that the color of OM is gray to off-white, consistent with the observations of oil-immersed whole-rock slices^84^. The sizes of the organic particles are mostly relatively large, ranging from 10 to 100 μm, and the shapes of the grains are generally flocculent, clumpy aggregates, elliptical and platy, potentially indicating a sapropelinite origin^85,86^. Due to the relatively strong heterogeneity of OM, its surface after ion polishing was relatively inhomogeneous. The selected indentation positions were the smoothest, with relatively compact and uniform OM distributions. The representative residual imprint of OM can be observed at Easy*R*_o_=1.04% (Fig. S2c).

**Figure 5:**
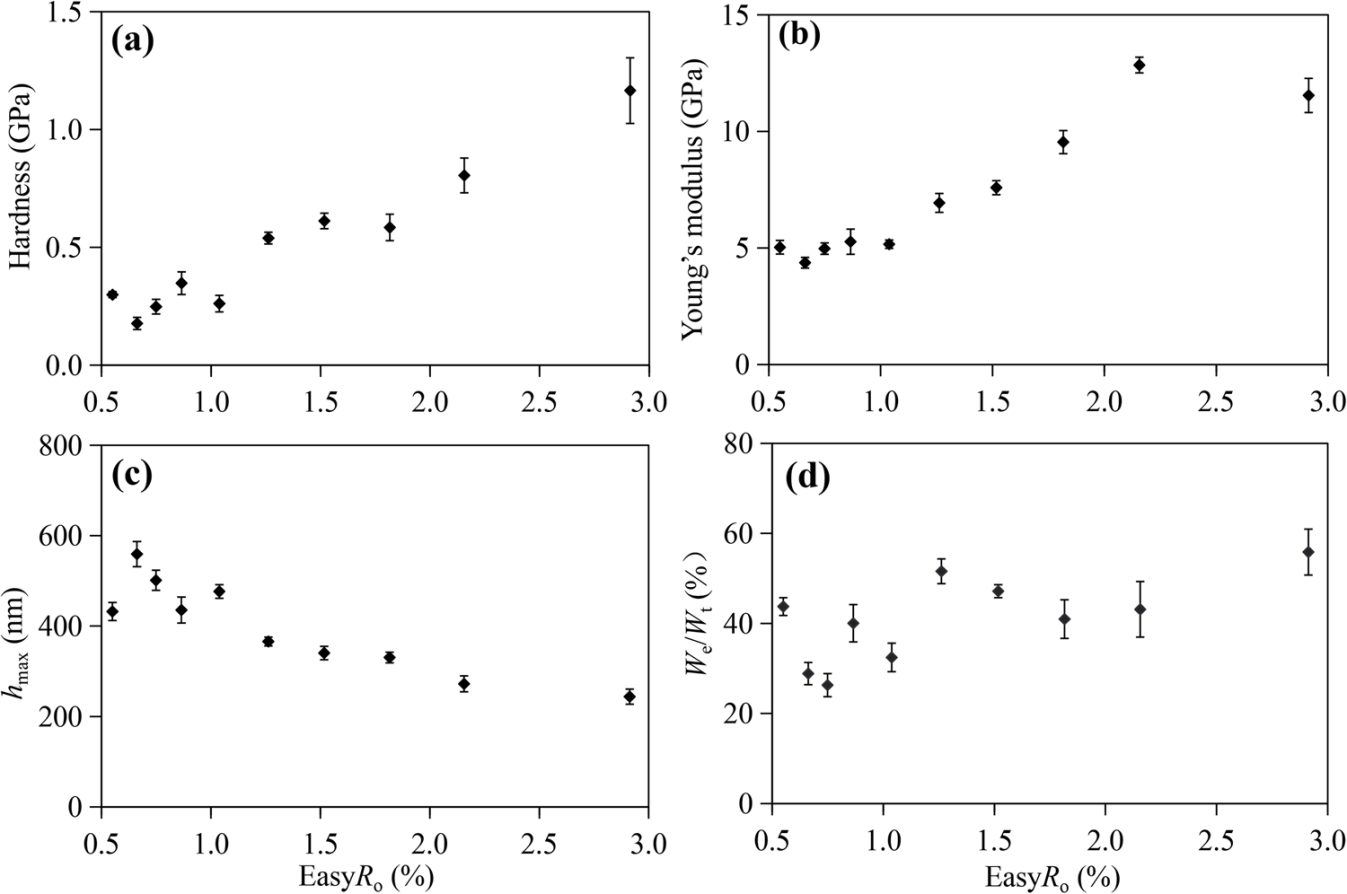
Plots of hardness (**a**), Young’s modulus (**b**), *h*_max_ (**c**) and *W*_e_/*W*_t_ (**d**) of the OM with respect to thermal maturity (Easy*R*_o_%). The error bars represent the standard deviation of the average value obtained from at least 9 repeated tests.

For the simulated shale samples, the variation of Young’s modulus, hardness, *h*_max_, and *W*_e_/*W*_t_ ratio as a function of thermal maturity is shown in Fig. 5. The displacement in the load-displacement curve (Fig. S3) for OM decreases with increasing maturity, with mean *h*_max_ values decreasing from 559.2 ± 77.3 nm at Easy*R*_o_=0.66% to 243.9 ± 24.9 nm at Easy*R*_o_=2.91% (Fig. 5c; Table S4). This is due to the increasing stiffness of the OM. The *W*_e_/*W*_t_ ratios (the elastic part of indentation work) generally show an increasing trend with maturity, although some samples exhibit exceptions. Mean *W*_e_/*W*_t_ ratios increase from 28.9% ± 3.7% at Easy*R*_o_=0.66–55.9% ± 5.0% at Easy*R*_o_=2.91%, suggesting that OM in shale gradually changes from plastic deformation to elastic deformation (Fig. 5d; Table S4). However, for the original shale sample, it exhibits similar *h*_max_, and mean *W*_e_/*W*_t_ ratio values with shale at Easy*R*_o_=0.87%.

The evolution of mechanical properties with maturity can be divided into two stages: the main oil-generation stage (Easy*R*_o_ ≤ 1.04%) and the condensate oil and gas generation stage (Easy*R*_o_ > 1.04%). During the main oil-generation stage, the Young’s modulus and hardness increase gradually, with the former ranging from 4.37 ± 1.07 to 5.16 ± 0.57 GPa and the latter from 0.18 ± 0.05 to 0.26 ± 0.03 GPa (Fig. 5a and b; Table S4). During the second stage, both the Young’s modulus and hardness of OM increase rapidly during the gas stage, from 6.93 ± 1.41 to 11.55 ± 2.25 GPa, and from 0.54 ± 0.25 to 1.17 ± 0.26 GPa, respectively (Fig. 5a and b; Table S4).

#### Mechanical properties of clay matrix

Figure S4 show the curves of load versus displacement of the typical shale sample at Easy*R*_o_=0.66% and 2.91% for peak load of 4.8 mN, respectively. The displacement is ranging from approximately 200 nm to 1000 nm at Easy*R*_o_=0.66%, while it varies from ~ 150 to 800 nm at Easy*R*_o_=2.91%. The mean displacement of the typical shale sample at Easy*R*_o_=0.66% and 2.91% is 679.8 ± 220.5 nm and 4623.0 ± 149.3 nm, respectively. The “pop in” event, characterized by a sudden jump of displacement during the loading stage, is observed in both samples. This is caused by the crack initiation or the action on the micro- or nano-scale pores and microfractures in the shale during the experiment^49,52^.

**Figure 6:**
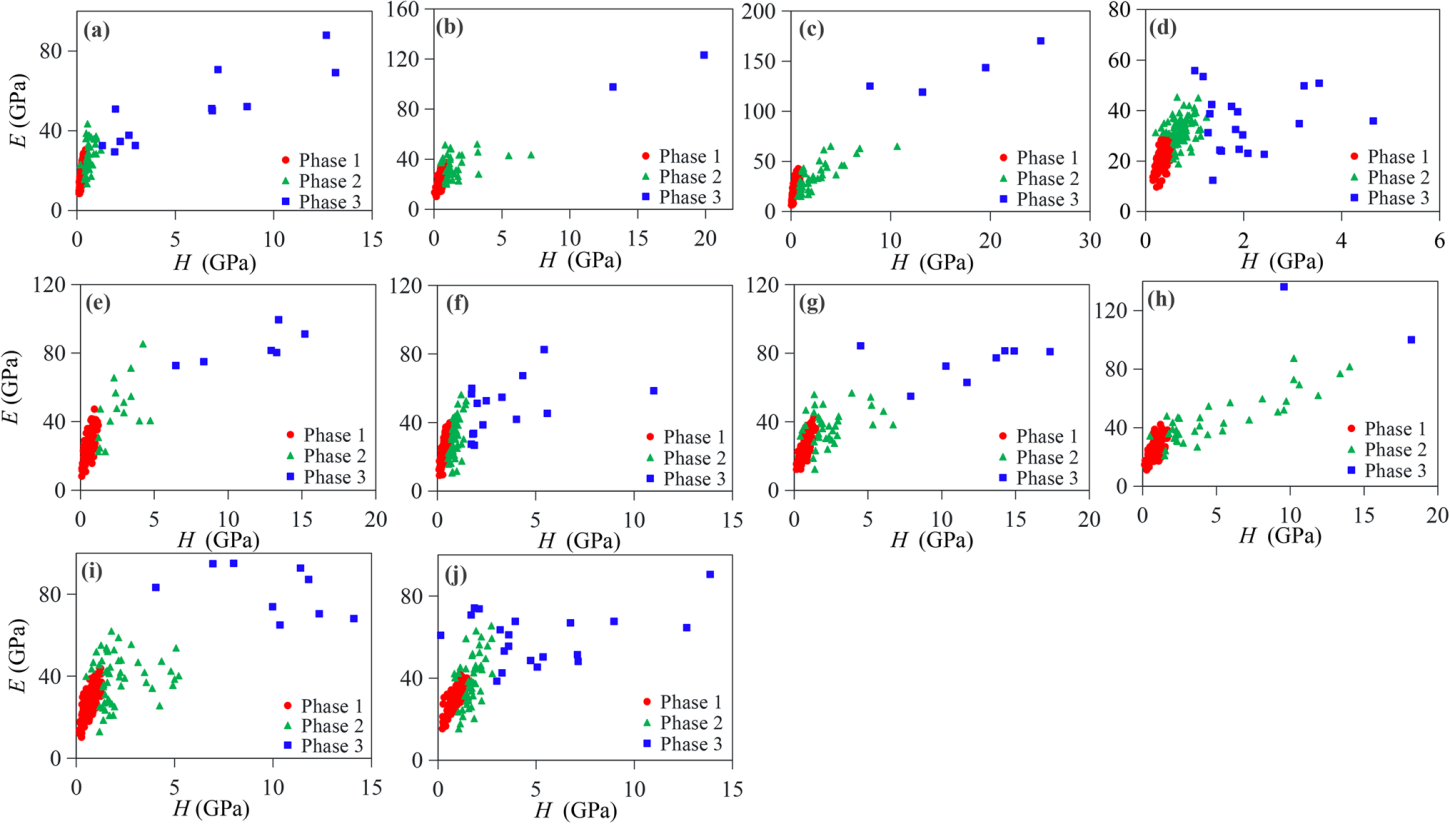
Clustering analysis results derived from indentation data of shales, arranged in order of increasing maturity (Easy*R*_o_%) (a:0.55%, b:0.66%, c:0.75%, d:0.87%, e:1.04%, f:1.26%, g:1.52%, h:1.82%, i:2.16%, j:2.91%). The inputs to the clustering analysis include *E* and *H*. (**a**) The *E*-*H* plot provides a top view of the Gaussian distribution across three phases: Phase 1 represents the clay matrix, while Phase 2 includes minerals containing calcite, clay-carbonate interfaces, clay-silicate interfaces, and boundaries of hard minerals. Phase 3, with the largest Young’s modulus, contains the hard minerals, such as quartz, feldspar, dolomite, and pyrite.

Figure 6 and Table S5 show the deconvolution results for Young’s moduli and hardness of all the samples. The mechanical properties of shales vary widely, with Young’s modulus ranging from ~ 5 to 150 GPa and hardness varying from ~ 0.1 to 20 GPa. According to the distribution of these mechanical properties and XRD results of mineralogy, three distinct mineral phases can be identified: the soft, complex and hard mineral phases (as shown in Fig. 6). For the heated shales at various maturities, the indentation response of the first phase has a mean Young’s modulus ranging from 22.6 to 29.8 GPa, the second phase from 31.8 to 45.1 GPa, and the third phase from 34.6 to 139.6 GPa (Table S3). According to the mineralogy of shales, the first phase with the lowest Young’s modulus and is likely to be the clay minerals and/or OM, i.e., clay matrix, the second phase is the complex mineral phases that contain calcite, clay mineral-silicate interfaces, clay minerals-carbonates interfaces, or the boundaries of hard minerals. The third phase is the hardest component contains the hard minerals, such as feldspar, quartz, dolomite, and pyrite. For all the shale samples, phase 1 is the largest volume fraction by deconvolution results, varying from 62.0 to 84.8% of the probed samples, which is relatively close to the XRD results except shale at Easy*R*_o_=0.87% and 2.91%. The deviation for these two shale samples may be due to the differences in the microscopic indentation areas of the shale. For the original shale sample, it has the similar phase distribution while having a lower Young’s modulus and hardness for each phase, which may be that it has not experienced the heated process and owned much water within shale. Overall, this result suggests that the clay matrix is the main phase in these shale samples.

**Figure 7:**
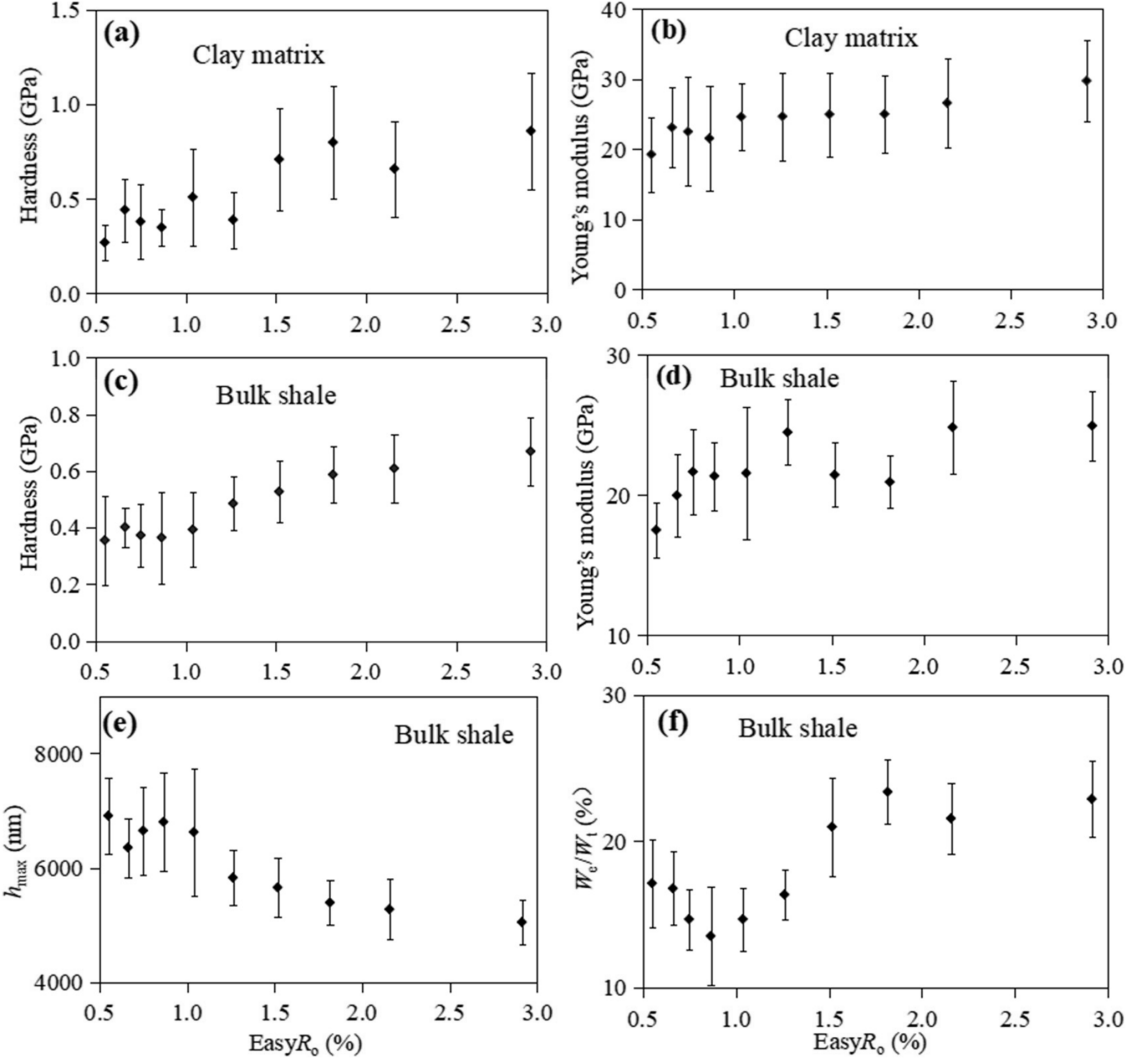
Plots of hardness (**a**) and Young’s modulus (**b**) of clay matrix with respect to thermal maturity (Easy*R*_o_%); hardness (**c**), Young’s modulus (**d**), *h*_max_ (**e**) and *W*_e_/*W*_t_ (**f**) of bulk shale with respect to thermal maturity (Easy*R*_o_%).

Overall, the mean Young’s modulus and hardness values of clay matrix exhibit an upward trend from Easy*R*_o_=0.66–2.91%, from 0.44 to 0.86 GPa (Fig. 7a), and from 23.1 to 29.8 GPa (Fig. 7b), respectively. Nevertheless, it decreased slightly between Easy*R*_o_=0.66% and Easy*R*_o_=1.04%. With increasing maturity, the mean Young’s modulus and hardness values of phase 2 vary relatively slightly, while the phase 3 exhibits a much wider range. This can be attributed to the fact that the indentations may be located on individual hard minerals, such as dolomite or pyrite, as evidenced by several indentations corresponding to phase 3 in Fig. 6b, c, and h.

#### Mechanical properties of bulk shale

The displacement ranges approximately 4000 nm to 8000 nm, with mean displacement of 6352.8 ± 611.9 nm at Easy*R*_o_=0.66%, while it ranges from approximately 4000 nm to 6000 nm with mean displacement of 5048.9 ± 399.4 nm at Easy*R*_o_=2.91% for the bulk shale. We observe a significantly higher occurrence of “pop in” behavior at the peak load of 350 mN compared to the peak load of 4.8 mN (Fig. S4), which is due to the different volumes and areas acted upon the shale by the indentation. Figure S4e and f show optical microscopy images of the shale samples of the load of 350 mN at Easy*R*_o_=0.66% and 2.91% after nanoindentation test, respectively. The size of indents is larger at Easy*R*_o_ of 0.66% than the ones at Easy*R*_o_ of 2.91%. A detailed analysis of the indents will be presented later based on the SEM results.

Consistent with the varying trend of the clay matrix with increasing maturity, the mean Young’s modulus and hardness values of bulk shale increase with increasing maturity (Fig. 7c and d). From Easy*R*_o_=0.66–2.91%, the hardness and Young’s modulus of bulk shale increase from 0.40 ± 0.07 GPa to 0.67 ± 0.12 GPa, and from 20.0 ± 3.0 GPa to 24.9 ± 2.5 GPa, respectively (Fig. 7). However, the hardness decreases slightly between Easy*R*_o_=0.66% and Easy*R*_o_=1.04%, consistent with the clay matrix, suggesting the oil generation of shale may have a much greater effect on the hardness value. The mean *h*_max_ values decrease from 6352.8 ± 611.9 nm at Easy*R*_o_=0.66% to 5048.9 ± 399.4 nm at Easy*R*_o_=2.91% (Fig. 7e), as the shale becomes increasingly stiff. Mean *W*_e_/*W*_t_ ratios increase from 16.8% ± 2.5% at Easy*R*_o_=0.66–22.9% ± 2.6% at Easy*R*_o_=2.91% (Fig. 7f), suggesting that the shale has more elastic deformation with increasing maturity, which is consistent with the OM. The original shale sample exhibits lower Young’s modulus and hardness, which may be also related to the large water content within shale.

## Discussion

### Influence factors on mechanical properties of OM in shale

The evolution of the Young’s modulus of OM with maturity has been previously reported by atomic force microscopy^23,24,30,87^ and nanoindentation^15,68^. While the OM is pure kerogen and solid bitumen extracted from the shale rock by the thermal simulation experiment^30,68^, or using the shale samples in a limited maturity range^15,23,24,87^. We studied the evolution of mechanical properties for OM in shale during the whole thermal evolution stage. This evolution is generally manifested in an increase in Young’s modulus. The Young’s modulus increases through the oil window and is the highest at the gas-generation stage, which has a maximum of 12.85 ± 1.16 GPa at Easy*R*_o_=2.16%. Although the Young’s modulus of OM increases with maturity, the increase is considerable, indicating a ~ 7 GPa increase throughout the entire thermal simulation experiment. However, during the main oil-generation stage, corresponding to Easy*R*_o_ ≤ 1.04%, the increase is relatively smaller, amounting to ~ 1 GPa. Therefore, we hypothesise that the evolution of mechanical properties of OM with maturity can be divided into the two stages: the main oil-generation stage (Easy*R*_o_ ≤ 1.04%) and the condensate oil and gas generation stage (Easy*R*_o_ >1.04%).

In the oil-generation stage, although f_al_ and Cn’ decrease with increasing maturity, the OM in shale has a large amount of aliphatic carbons and long-chain aliphatics. The aromatic layers are thin and sparsely distributed, and the stacking layers are sparse as well, so the molecular structure stability of OM at this stage is low and the resistance to indentation load is limited, resulting in low measured Young’s modulus and hardness values, which is consistent with the data on kerogen from simulated experiments^68^. Besides, the C-C, C-N and C-S bonds and some of the C-O bonds are broken, and the hydrocarbon side chains are lost^88^, which will produce the gas and liquid hydrocarbons. The adsorption of liquid hydrocarbons by OM may contribute to its mechanical properties remaining relatively unchanged during this stage.

In contrast, OM becomes much stiffer as maturation progresses to the condensate oil and gas generation stage. Most of the long-chain aliphatic carbons in OM have been lost or transformed into short-chain gas hydrocarbon products (< C_5_) through the heating process^61,88^. This leads to a more ordered, aromatic carbon enrichment, higher shrinkage of aromatic layer, and larger number of stacked layers structure^89^, exhibited by the high f_ar_ and X_BP_ values in the NMR results (Fig. 4; Table S3). Therefore, OM has the higher molecular structure stability and can resist the load applied to its surface, the Young’s modulus and hardness of OM become increasingly larger.

**Figure 8:**
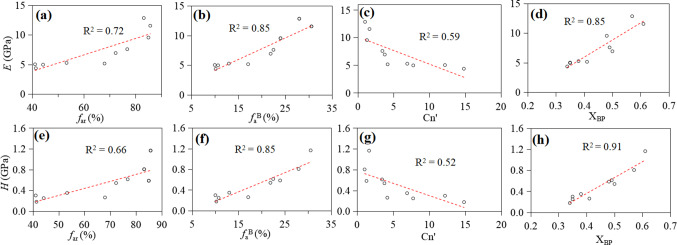
Plots of Young’s modulus (*E*) versus *f*_ar_ (**a**), *f*_a_^B^ (**b**), Cn’ (**c**) and X_BP_ (**d**); hardness versus *f*_ar_ (**e**), *f*_a_^B^ (**f**), Cn’ (**g**) and X_BP_ (**h**) of OM.

**Figure 9:**
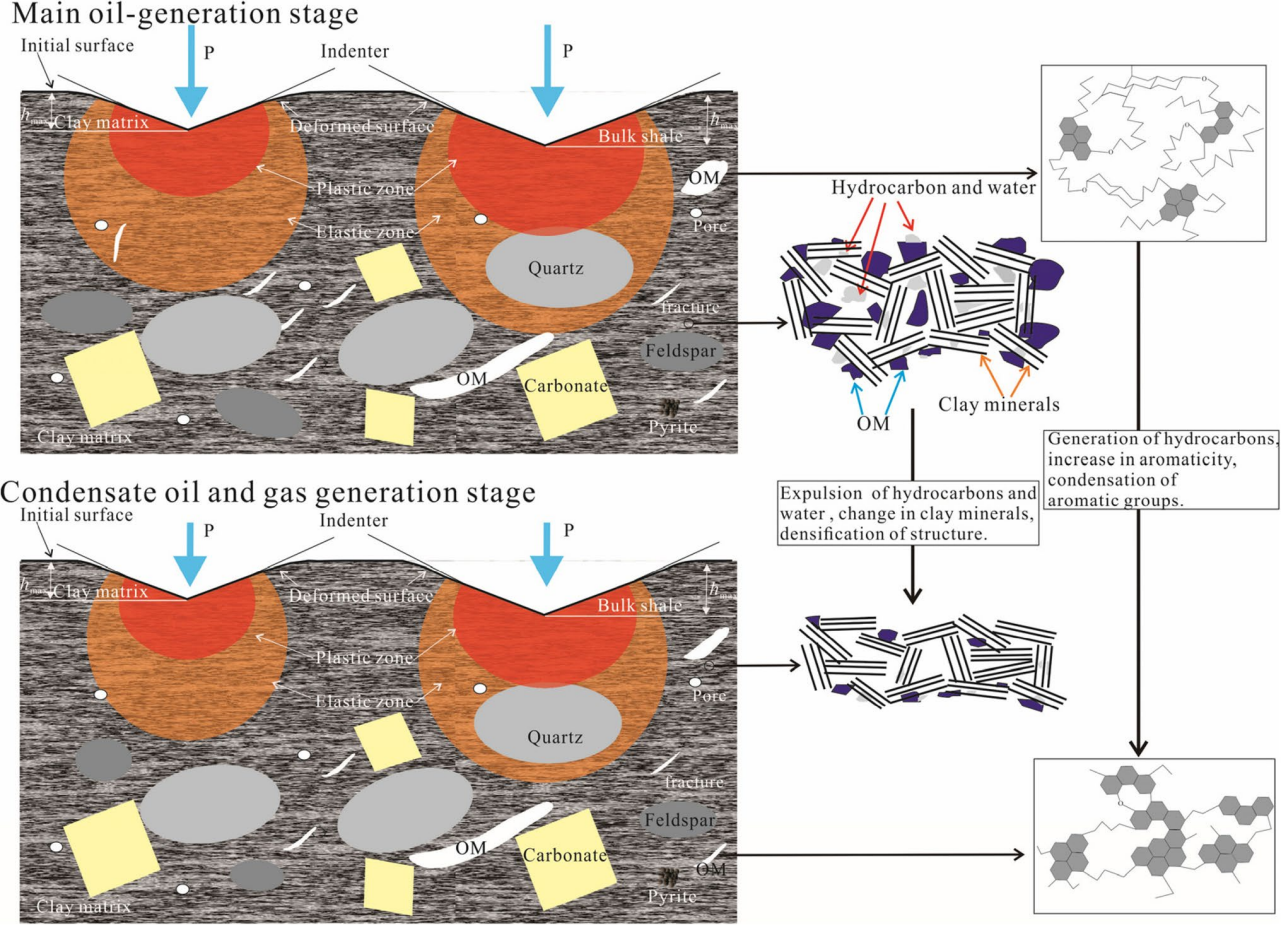
Schematic diagram of nanoindentation of shale samples at oil- and gas-window maturity. Representation of the structure of organic matter (OM) and clay matrix (to the right of the arrow) at the main oil-generation stage (upper part) and the condensate oil and gas generation stage (lower part).

The overall increase in hardness and Young’s modulus of OM with maturity largely results from the variation in its chemical structure, as suggested by the good correlation between the two parameters (Fig. 8). The good positive correlation between hardness, Young’s modulus, and f_ar_ (R^2^ > 0.66; Fig. 8) shows that increasing aromaticity in the organic structures contributes partly to the increase in mechanical parameters. There is a strong correlation between mechanical parameters and f_a_^B^ (R^2^ = 0.85). This suggests that the increase of f_a_^B^ may be the reason to cause the increase of mechanical parameters. The significant negative correlation between mechanical parameters and Cn’ (R^2^ > 0.52; Fig. 8) shows that the short length of aliphatic chains in organic structures also enhance mechanical parameters. The better correlation between mechanical parameters and X_BP_ (R^2^ ≥ 0.85) indicates that increasing condensation may also contribute partly to the increase of mechanical parameters. Overall, a more ordered, aromatic carbon enrichment, higher shrinkage of aromatic layer, and a greater number of stacked layers structure^89^ are all the reasons for the increase in mechanical parameters for OM (Fig. 9).

### Influence factors on mechanical properties of clay matrix

The hardness and Young’s modulus of clay matrix decreased slightly between Easy*R*_o_=0.66% and Easy*R*_o_=1.04%, which may be attributed to the generation of significant amounts of oil within this maturity range, as verified by the S_1_ (mean free hydrocarbon content in the rock) and S_2_ (the total hydrocarbons generated through thermal cracking of nonvolatile organic matter) values in Table S1. These liquid hydrocarbons can exist in clay matrix in adsorbed or free state, potentially inhibiting the increase of elastic modulus during maturation. Although the strengthening of OM in the clay matrix may lead to an increase in mechanical parameters, the extent of this increase may be offset by the presence of residual hydrocarbons within the clay matrix. However, as maturity increases further, the mechanical parameters of the clay matrix generally increase. This trend is primarily due to the dehydration of shale, the release or decomposition of liquid hydrocarbons, and the transformation of clay minerals (Fig. 9).

**Figure 10:**
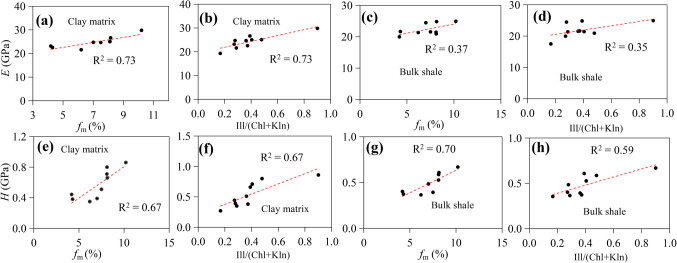
Plots of Young’s modulus (*E*) versus *f*_m_ for both clay matrix (**a**) and bulk shale (**c**), Young’s modulus (*E*) versus the ratio of illite to (chlorite + kaolinite) content for both clay matrix (**b**) and bulk shale (**d**). Hardness (*H*) versus *f*_m_ for both clay matrix (**e**) and the bulk shale (**g**), Hardness (*H*) versus the ratio of illite to (chlorite + kaolinite) content for both clay matrix (**f**) and bulk shale (**h**).

Figure 10a-b and e-f show the relationship between mechanical parameters and the mass loss rate of shale (f_m_) or the ratio of illite to (chlorite + kaolinite) content during thermal maturation. When the shale is heated, the different states of water, i.e., free water, adsorbed water and crystal water in clay matrix, will escape. When the temperature reaches about 100℃, the free water is basically completely removed, adsorbed water and the structural water can evaporate completely at approximately 100–300 ℃ and 300–500 °C, respectively^26,90^. Due to our simulated experiment is being conducted above 300 °C, most water in clay will evaporate, the intermolecular spaces in the clay gradually shrink and the intermolecular attraction increases, making the overall clay structure more firm and compact, eventually leading to clay hardening^26^. Also, evaporation of water reduces pore pressure in pores, increases effective stress, and leads to mutual compression of mineral particles^91^, thus improving the strength of clay matrix in shale. This can also be verified from the positive relationship between mechanical parameters and the mass loss rate of shale (f_m_) (R^2^ ≥ 0.67) (Fig. 10a and e). During the indentation process, clay matrix exhibits increased elastic deformation, resulting in a smaller h_max_ value, as well as a larger Young’s modulus and hardness with increasing maturity (Fig. 9).

Besides, the transformation of kaolinite to illite may also contribute to the hardening of clay matrix. The transformation process is as follows^92,93^:

1 $$\mathrm{Al}2\mathrm{Si}2\mathrm O5(\mathrm{OH})4(\mathrm{kaolinite})+\mathrm{KAlSi}3\mathrm O8(\mathrm K-\mathrm{feldspar})=\mathrm{KAl}3\mathrm{Si}3\mathrm O10(\mathrm{OH})2(\mathrm{illite})+2\mathrm{SiO}2+\mathrm H2\mathrm O$$

2 $$3\mathrm{KAlSi}3\mathrm O8(\mathrm K-\mathrm{feldspar})+2\mathrm H++\mathrm H2\mathrm O=\mathrm{KAl}3\mathrm{Si}3\mathrm O10(\mathrm{OH})2(\mathrm{illite})+6\mathrm{SiO}2(\mathrm{silicatemineral})+2\mathrm K++\mathrm H2\mathrm O$$

The conversion of kaolinite to illite will consume K-feldspar (Table S2), resulting in its dissolution and subsequent reduction in content, and then generate siliceous minerals, which exist in the form of quartz secondary enlargement or cement^94^. This, in turn, enhances the compaction resistance of the clay matrix. This transformation process can be ensured by the relatively good linear correlation between the ratio of illite to (chlorite + kaolinite) content and both Young’s modulus (R^2^ = 0.73) (Fig. 10b) and hardness (R^2^ = 0.67) (Fig. 10f).

**Figure 11:**
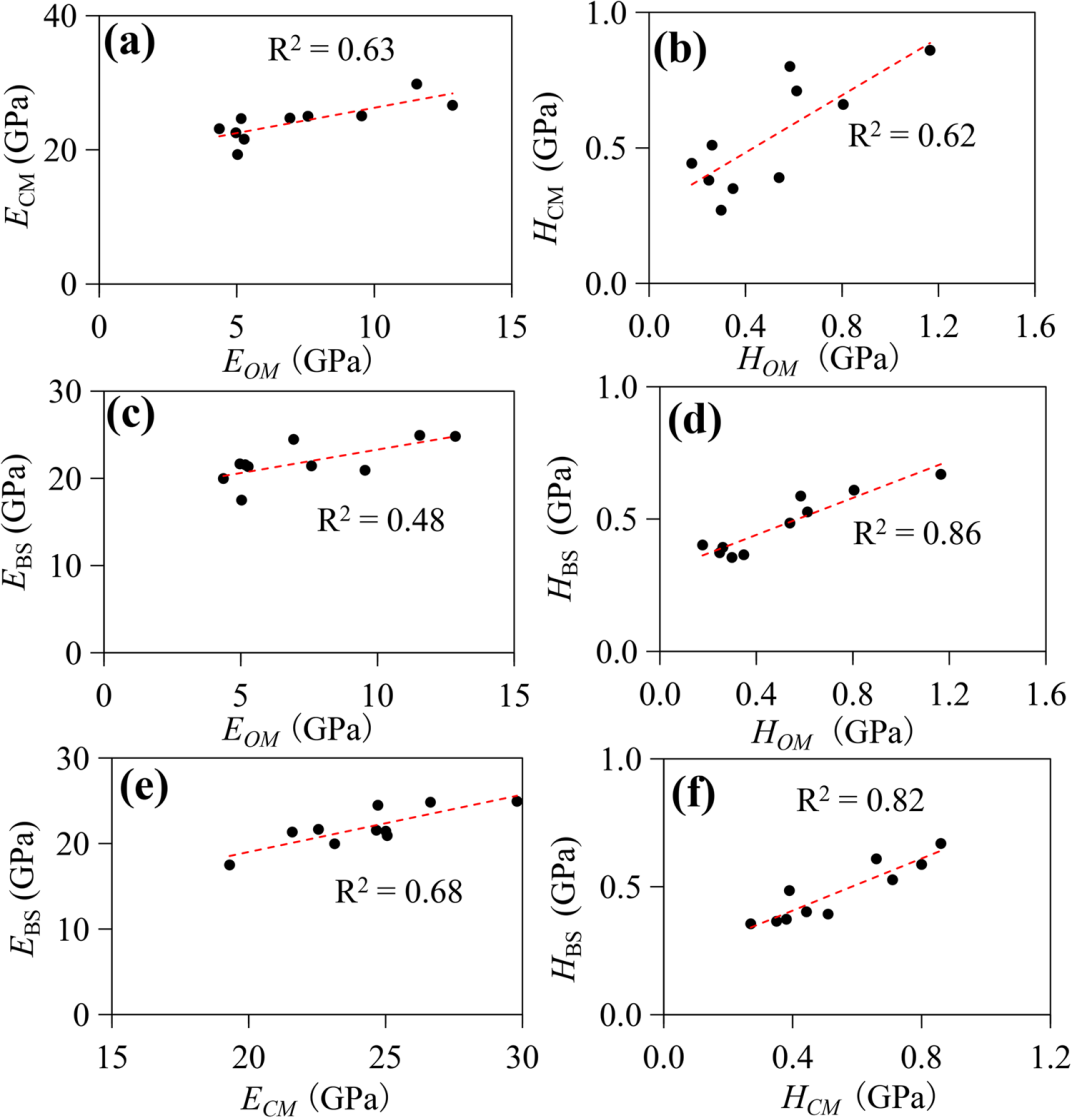
Relationships between *E*_CM_ and *E*_OM_ (**a**), *H*_CM_ and *H*_OM_ (**b**); *E*_BS_ and *E*_OM_ (**c**) and *H*_BS_ and *H*_OM_ (**d**), *E*_BS_ and *E*_OM_ (**e**) and *H*_BS_ and *H*_CM_ (**f**). Young’s modulus of organic matter (*E*_OM_ ), clay matrix (*E*_CM_) and bulk shale (*E*_BS_); hardness of organic matter (*H*_OM_), clay matrix (*H*_CM_) and bulk shale (*H*_BS_).

On the other hand, due to the clay matrix containing amounts of OM, the decrease of OM (i.e., the softest component in shale) and the expulsion or cracking of liquid hydrocarbons at the high simulated temperature may partly lead to the increase in Young’s modulus and hardness. Besides, the hardening of OM with maturation may also contribute to the increase in mechanical parameters of the clay matrix. This relationship can be verified by the good positive correlation between OM and the clay matrix in terms of both Young’s modulus (R^2^ = 0.63) and hardness (R^2^ = 0.62), as depicted in Fig. 11a and b.

### Influence factors on mechanical properties of bulk shale

Similar to the clay matrix, the mean hardness values of bulk shale decrease before Easy*R*_o_=1.04%, then it generally increases with increasing maturity (Fig. 7c and d). This decreasing trend can also be attributed to the oil generation of shale during this maturity range. The variation of Young’s modulus of bulk shale with maturity is generally consistent with hardness. However, it shows a downward trend between Easy*R*_o_=1.52% and 1.82%. We hypothesize that the appearance of induced pores and fractures would decrease the stiffness of the bulk shale^69^.

For the variation of micro-mechanical parameters (i.e., Young’s modulus and hardness) of bulk shale with maturity, previous studies have showed that they first decrease and then increase, which is mainly due to the change of pore structures in shale and the structure of OM during pyrolysis^70^. However, Zhang, et al.^69^ found Young’s modulus and hardness show an opposite trend as the maturity value increases, which is also related to the stiffening of OM and the appearance of induced pores and fractures during maturation. However, our results are not entirely consistent with the above findings. Considering the similar heating temperature, we believe the differences in TOC and mineral contents among various shale types may account for the varying results^20,57,95^. Under high temperature, water loss causes mineral particles to expand and compress more closely together, while the evaporation of adsorbed water reduces the lubrication effect during mineral particle sliding^26^ (Fig. 9). When subjected to indentation loads, mineral particles are less prone to rearrangement, and the primary pore structure collapses to a certain extent, leading to an increase in elastic modulus. This observation is supported by the positive correlation between mechanical parameters and the mass loss rate of shale (f_m_) for bulk shale samples (Fig. 10c and g). Additionally, water loss in clay can trigger the transformation of kaolinite to illite, hardening the clay matrix and further enhancing the stiffness of bulk shale^26,90^. This is corroborated by the positive linear correlation between the ratio of illite to (chlorite + kaolinite) content and hardness or Young’s modulus (Fig. 10d and h). In addition, the hardening of OM or clay matrix with maturation may also contribute to the increase of mechanical parameters, which can be verified by the good positive relationship for Young’s modulus and hardness between the bulk shale and OM or clay matrix (Fig. 11c −f).

Based on the analysis of indents from Fig. S5, representative images were chosen from the samples at Easy*R*_o_=0.87% and 2.91%. The phase distributions of the whole indentation regions (~ 700 × 700 μm^2^) in the shale surface are all clay-rich areas (Fig. S5). The various regions within an identical sample demonstrate similar mechanical characteristics. Although all the indents in the two samples at Easy*R*_o_=0.87% and 2.91% are almost located in the clay-rich area, the indent located in the area with relatively high OM (Fig. S5d’) has the lower mechanical parameters at Easy*R*_o_=0.87%, and that with relatively high quartz has larger mechanical parameters at Easy*R*_o_=2.91% (Fig. S5f’). The indentation impression for the shale at Easy*R*_o_=0.87% has a radius of 28.2–32.6 μm, while the radius of the other shale sample at Easy*R*_o_=2.91% is 19.7–23.2 μm. This suggests the shale with high maturity has a more compact and dense microstructure and has a greater resistance to indentation loads (Fig. 9), which can be confirmed by the larger mechanical parameters in Figs. S5f-5 h than those in Figs. S5b-5d. The indents for mechanical analysis also explain the reason that the bulk shale becomes increasingly stiff with increasing maturity.

## Conclusions

This study investigates the evolution of the mechanical properties of Yanchang Formation shale and the controlling factors during the maturation process at the nano- to micro-scale. It is found that the Young’s modulus and hardness values of bulk shale typically exhibit an increasing trend with increasing maturity, which can be attributed to the stiffening of OM and clay matrix during maturation, as well as the development of a more compact and dense microstructure. Moreover, the OM shows a slight increase in hardness and Young’s modulus at Easy*R*_o_ ≤ 1.04%. With increasing maturity, these mechanical parameters increase significantly, mainly due to the variations in chemical structures. The mechanical parameters of the clay matrix decrease slightly at first and then generally show an increasing trend with increasing maturity. This can be mainly attributed to the dehydration of shale, the expulsion or cracking of liquid hydrocarbons, the transformation of clay minerals, and the hardening of OM.

Such quantitative measurements of the mechanical properties of OM, clay matrix, and bulk shale during thermal maturation can serve as model inputs for reservoir development and provide fundamental data for engineering applications in the in-situ conversion of shale oil. These observations have furthered our understanding of the role of OM and clay matrix in determining the evolution of bulk shale mechanical properties, and in particular, shed light on the future accurate evaluation of sweet spots for shale oil exploration and development in the Chang 7 shale of the Upper Triassic Yanchang Formation in China. However, the effects of fluid (e.g., H_2_O) and confining pressure were not considered in this study and we conducted experiments on the artificial maturation process of shale. Future research should consider the characteristics of in-situ shale reservoir conditions, and compare the artificially matured shale with the naturally matured shale samples.

## Supplementary Information

Supplementary Material 1.

## Data Availability

The datasets used and/or analyzed during the current study are available from the corresponding author on reasonable request.
