# Supplementary material for: Evolution of mechanical properties of organic-rich shale during thermal maturation
Source: Sci Rep. 2024 Oct 17;14:24327. doi: 10.1038/s41598-024-75035-7 (PMC11484891; doi:10.1038/s41598-024-75035-7)
Supplement: Supplementary file 1 — Supplementary Material 1. [file 41598_2024_75035_MOESM1_ESM.docx]

Supplementary Information for

**Evolution of mechanical properties of** **organic-rich shale** **during** **thermal maturation**

The supplementary material contains five figures and five tables

Jianfeng Wang ^a,b, c,d^, Dayong Liu ^a,b^*, Jianfei Shi ^a,b^, Chao Yang ^e^, Yuke Liu ^f^, Guozhi Wang ^a,b^, Huijuan Guo ^a,b^, Peng Liu ^g^, Yongqiang Xiong ^a,b^**, Ping’an Peng ^a,b^

a. State Key Laboratory of Organic Geochemistry, Guangzhou Institute of Geochemistry, Chinese Academy of Sciences, Guangzhou 510640, China

b. CAS Center for Excellence in Deep Earth Science, Guangzhou 510640, China

c. Key Laboratory of Petroleum Resources Research, Gansu Province, Lanzhou 730000, China

d. Research Center for Oil and Gas Resources, Northwest Institute of Eco-Environment and Resources, Chinese Academy of Sciences, Lanzhou 730000, China

e. Key Laboratory of Natural Gas Hydrate, Guangzhou Institute of Energy Conversion, Chinese Academy of Sciences, Guangzhou 510640, China

f. Research Institute of Petroleum Exploration and Development, Beijing 100083, China

g. College of Safety Science and Engineering, Xi’an University of Science and Technology, Xi’an, 710054, Shanxi, China

* Corresponding Authors: Dayong Liu;liudayong@gig.ac.cn

Yongqiang Xiong; [xiongyq@gig.ac.cn](mailto:xiongyq@gig.ac.cn)

.


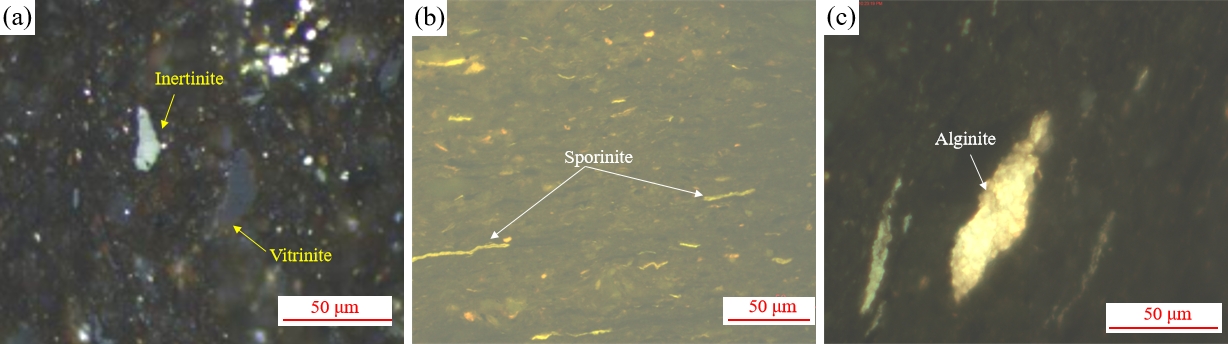


Figure S1. Petrographic identification of organic components in the original Yanchang Formation shale. (a) Vitrinite and inertinite under oil-immersion reflected light; (b) Sporinite under fluorescence light; (c) Alginite under fluorescence light.


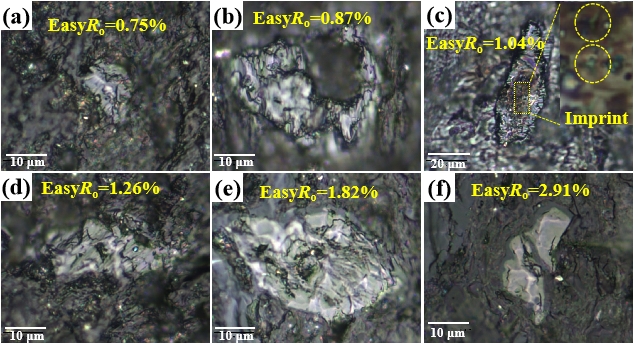


Figure S2. The typical optical microscope images taken by a nanoindenter at indentation sites on organic matter in shale, shown in order of increasing maturity (Easy*R*_o_%) (a:0.75%, b:0.87%, c:1.04%, d:1.26%, e:1.82%, f:2.91%). (c) Residual indentation imprints after indentation in the surface of the organic matter can be observed.

Figure S3. Typical load-displacement curves of the organic matter in shale with respect to maturity (Easy*R*_o_%).


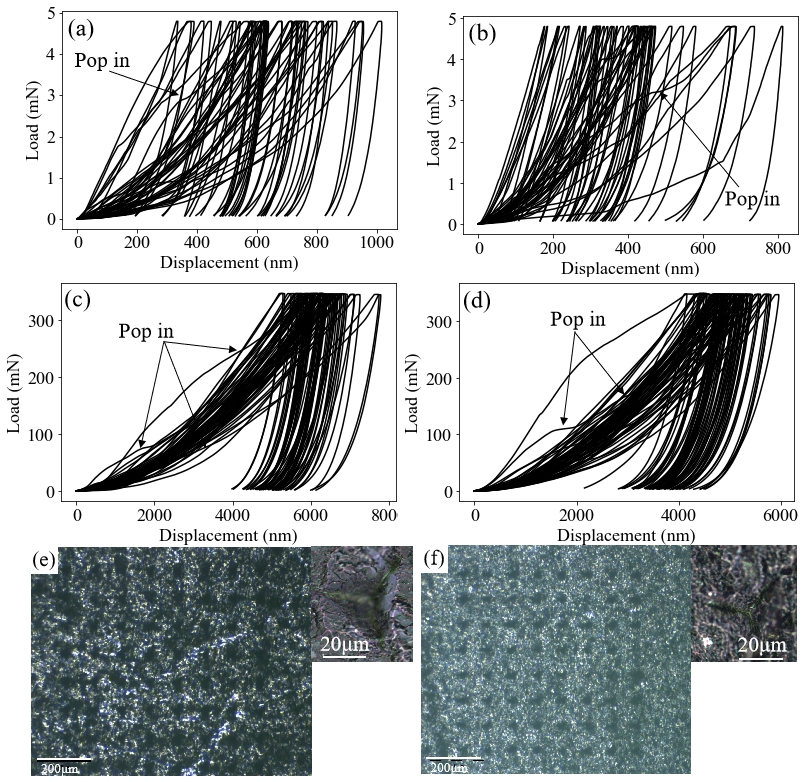


Figure S4. Typical curves of load versus displacement of the shale from the load of 4.8 mN at Easy*R*_o_ of 0.66% (a) and 2.91% (b) and from the load of 350 mN at Easy*R*_o_ of 0.66% (c) and 2.91% (d). (e) and (f) are the corresponding optical microscopy images of the load of 350 mN at Easy*R*_o_ of 0.66% and 2.91% after nanoindentation test, respectively. The typical imprint for both (e) and (f) is magnified and displayed in the right corner of the respective images.


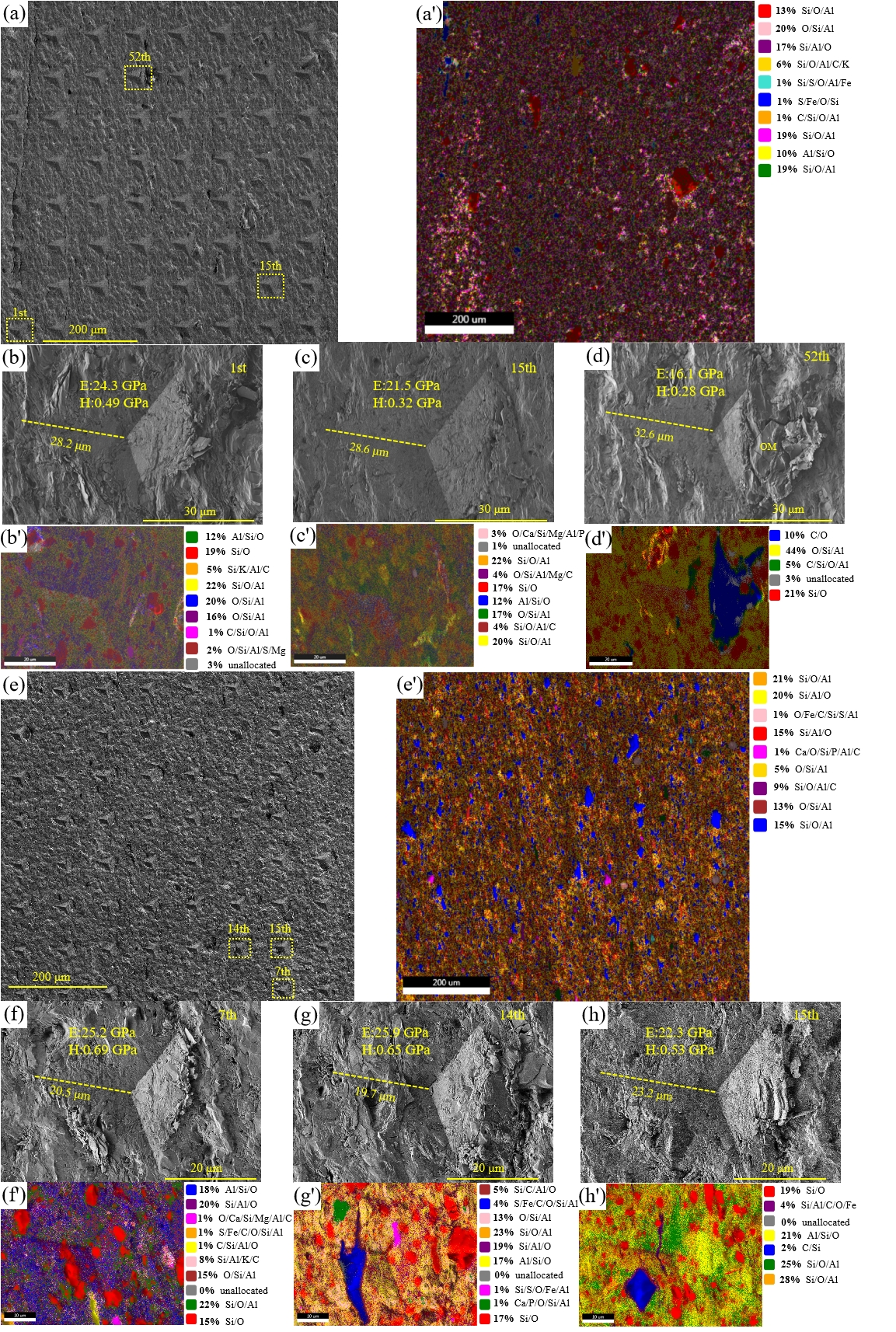


Figure S5. Backscattering (BSE) diagrams (a-h) and phase distribution diagram (a'-h') of indentation areas for shale (a-d and a'-d' at Easy*R*_o_=0.87%; e-h and e'-h' at Easy*R*_o_= 2.91%, respectively). The series numbers in (a) and (e) represent the selected indentations for mechanical analysis. Based on the phase distribution images (b'-d' and f'-h'), all the indentations are primarily located in the clay-rich areas. However, the indentation located in the area with relatively high organic matter (d') exhibits the lowest mechanical parameters, while the indentation in the area with relatively high quartz content (f') displays the largest mechanical parameters. The indentation radius for the shale at Easy*R*_o_=0.87% is 28.2-32.6 μm, whereas the indentation radius for the other shale sample at Easy*R*_o_=2.91% is 19.7-23.2 μm.

Table S1. Changes in mass and residual organic carbon, and Rock-Eval data for shale. N/A indicates data not available.

| Simulated temperature（℃） | Easy*R*_o_（%） | m1  (g) | m2  (g) | f_m_ (%) | TOC  (%) | f_RT_（%） | S_1_  (mg/g) | S_2_  (mg/g) | T_max_  （℃） | HI  (mg/g TOC) |
| --- | --- | --- | --- | --- | --- | --- | --- | --- | --- | --- |
| Original | 0.55 | N/A | N/A | N/A | 12.06 | N/A | 4.00 | 69.32 | 441 | 582 |
| 320 | 0.66 | 41.15 | 39.43 | 4.19 | 11.62 | 92.35 | 2.57 | 62.95 | 443 | 555 |
| 340 | 0.75 | 42.46 | 40.63 | 4.30 | 11.28 | 89.54 | 4.24 | 51.81 | 445 | 501 |
| 360 | 0.87 | 42.27 | 39.65 | 6.20 | 9.93 | 77.26 | 2.90 | 32.10 | 446 | 331 |
| 380 | 1.04 | 40.74 | 37.69 | 7.50 | 8.37 | 64.19 | 0.22 | 11.22 | 454 | 135 |
| 400 | 1.26 | 24.00 | 22.32 | 7.01 | 8.00 | 61.68 | 0.05 | 3.72 | 516 | 46 |
| 420 | 1.52 | 18.81 | 17.28 | 8.10 | 7.50 | 57.15 | 0.05 | 1.69 | 574 | 20 |
| 440 | 1.82 | 16.74 | 14.05 | 8.11 | 7.30 | 55.62 | 0.01 | 1.04 | 593 | 13 |
| 460 | 2.16 | 19.39 | 17.81 | 8.15 | 6.90 | 52.55 | 0.01 | 0.68 | 604 | 9 |
| 500 | 2.91 | 13.35 | 11.99 | 10.20 | 6.70 | 49.89 | 0 | 0 | 610 | 6 |

m1:mass of shale before heating

m2:mass of shale after heating

Mass loss rate of shale (f_m_):(m1-m2)/m1

f_RT_:the residual TOC of shale/the original TOC of shale×(1-f_m_)

S_1_: mean free hydrocarbon content in the rock

S_2_:the total hydrocarbons generated through thermal cracking of nonvolatile organic matter

T_max_: the temperature at which the S_2_ attains its maximum hydrocarbon generation

HI:S_2_/TOC×100

Table S2. Mineral content of shales in order of increasing maturity (Easy*R*_o_%)

| Simulated temperature (℃) | Easy*R*_o_ (%) | Qtz  (%) | Fsp  (%) | Ill  (%) | Chl  (%) | Kln  (%) | Py  (%) | Gy  (%) | Cal  (%) | Cly  (%) | Ill/(Chl+Kln) |
| --- | --- | --- | --- | --- | --- | --- | --- | --- | --- | --- | --- |
| Original | 0.55 | 14.8 | 6.0 | 11.0 | 15.2 | 50.6 | 1.7 | 0.6 | - | 76.8 | 0.167 |
| 320 | 0.66 | 15.7 | 3.5 | 17.2 | 14.6 | 48.6 | 0.5 | - | - | 80.4 | 0.272 |
| 340 | 0.75 | 15.7 | 3.0 | 22.0 | 13.6 | 45.4 | 0.3 | - | - | 81.0 | 0.373 |
| 360 | 0.87 | 17.0 | 3.0 | 17.9 | 14.3 | 47.7 | - | - | - | 79.9 | 0.289 |
| 380 | 1.04 | 13.2 | 2.5 | 22.4 | 14.3 | 47.6 | - | - | - | 84.3 | 0.362 |
| 400 | 1.26 | 14.4 | 2.6 | 18.1 | 15.0 | 49.9 | - | - | - | 83.0 | 0.279 |
| 420 | 1.52 | 13.1 | 1.6 | 24.6 | 14.0 | 46.7 | - | - | - | 85.3 | 0.405 |
| 440 | 1.82 | 18.7 | 2.2 | 25.6 | 12.3 | 41.2 | - | - | - | 79.1 | 0.479 |
| 460 | 2.16 | 14.9 | 2.8 | 23.1 | 13.6 | 45.5 | - | - | - | 82.2 | 0.391 |
| 500 | 2.91 | 16.3 | 2.7 | 37.2 | 9.5 | 31.8 | - | - | 2.6 | 78.5 | 0.901 |

Note:Quartz=Qtz; Feldspar=Fsp; Illite=Ill; Chlorite=Chl; Kaolinite=Kln; Pyrite=Py; Gypsum=Gy; Calcite=Cal; Cly=Clay mineral

Table S3. Composition obtained from quantitative ^13^C CP-TOSS/MAS NMR spectra

| Easy*R*_o_（%） | Aliphatic（0 ppm~90 ppm，%） | | | | | | Aromatic（90 ppm~165ppm，%） | | | | f_al_% | f_ar_% | Cn' | X_BP_ |
| --- | --- | --- | --- | --- | --- | --- | --- | --- | --- | --- | --- | --- | --- | --- |
|  | f_CH3al_ | f_CH3ar_ | f_CH2_ | f_CH_ | f_C_ | f_O_ | f_a_^H^ | f_a_^B^ | f_a_^S^ | f_a_^O^ |  |  |  |  |
| 0.55 | 11.9 | 7.3 | 15.7 | 9.2 | 5.8 | 7.7 | 21.8 | 10.0 | 4.7 | 2.0 | 57.7 | 41.1 | 12.18 | 0.35 |
| 0.66 | 10.3 | 10.2 | 13.9 | 8.5 | 6.8 | 7.0 | 23.3 | 10.2 | 3.8 | 3.0 | 56.8 | 41.4 | 14.82 | 0.34 |
| 0.75 | 9.4 | 10.9 | 11.1 | 10.6 | 5.3 | 6.8 | 20.0 | 10.7 | 7.0 | 3.6 | 54.1 | 44.2 | 7.76 | 0.35 |
| 0.87 | 8.4 | 5.0 | 9.2 | 9.9 | 2.3 | 10.0 | 24.2 | 13.0 | 6.5 | 4.0 | 44.9 | 53.3 | 6.92 | 0.38 |
| 1.04 | 4.7 | 7.2 | 4.9 | 3.3 | 2.6 | 7.3 | 29.9 | 17.1 | 7.1 | 4.7 | 29.8 | 68.0 | 4.18 | 0.41 |
| 1.26 | 5.4 | 5.3 | 3.4 | 1.4 | 0.0 | 9.8 | 33.3 | 21.9 | 6.7 | 3.4 | 25.3 | 72.3 | 3.78 | 0.50 |
| 1.52 | 2.1 | 3.4 | 2.0 | 0.9 | 2.1 | 10.2 | 34.4 | 22.5 | 6.0 | 6.0 | 20.7 | 76.8 | 3.45 | 0.49 |
| 1.82 | 1.4 | 2.7 | 0.9 | 0.4 | 0.3 | 6.0 | 35.2 | 24.0 | 9.1 | 5.5 | 11.7 | 85.0 | 1.29 | 0.48 |
| 2.16 | 2.2 | 1.0 | 0.5 | 1.8 | 1.1 | 4.4 | 32.3 | 28.0 | 10.6 | 6.6 | 10.9 | 83.2 | 1.03 | 0.57 |
| 2.91 | 0.0 | 0.6 | 1.5 | 2.0 | 0.0 | 8.6 | 34.3 | 30.6 | 7.7 | 8.3 | 12.7 | 85.7 | 1.65 | 0.61 |

X_BP_: Ratio of bridgehead carbon to aromatic peripheral carbon.

Cn': Ratio of branched carbon to aliphatic carbon.

Table S4. Results of nanoindentation tests of the organic matter in shale.

| Simulated temperature（℃） | Easy*R*_o_（%） | *h*_max_ (nm) | | *W*e/*W*t (%) | | Hardness (GPa) | | Young's Modulus (GPa) | | Number of measurement |
| --- | --- | --- | --- | --- | --- | --- | --- | --- | --- | --- |
|  |  | Ave. | Std. | Ave. | Std. | Ave. | Std. | Ave. | Std. |  |
| Original | 0.55 | 432.3 | 41.7 | 43.7 | 5.7 | 0.30 | 0.07 | 5.03 | 0.71 | 12 |
| 320 | 0.66 | 559.2 | 77.3 | 28.9 | 3.7 | 0.18 | 0.05 | 4.37 | 1.07 | 9 |
| 340 | 0.75 | 501.4 | 83.4 | 26.3 | 3.3 | 0.25 | 0.08 | 4.97 | 1.31 | 9 |
| 360 | 0.87 | 435.5 | 79.3 | 40.0 | 7.7 | 0.35 | 0.11 | 5.27 | 0.69 | 14 |
| 380 | 1.04 | 476.5 | 42.3 | 32.4 | 3.0 | 0.26 | 0.05 | 5.16 | 0.57 | 18 |
| 400 | 1.26 | 365.9 | 68.8 | 51.6 | 14.7 | 0.54 | 0.25 | 6.93 | 1.41 | 18 |
| 420 | 1.52 | 340.6 | 58.2 | 47.2 | 8.5 | 0.61 | 0.29 | 7.59 | 1.90 | 14 |
| 440 | 1.82 | 330.6 | 52.5 | 41.0 | 9.6 | 0.58 | 0.21 | 9.55 | 2.34 | 15 |
| 460 | 2.16 | 272.3 | 25.9 | 43.1 | 7.6 | 0.81 | 0.20 | 12.85 | 1.16 | 18 |
| 500 | 2.91 | 243.9 | 24.9 | 55.9 | 5.0 | 1.17 | 0.26 | 11.55 | 2.25 | 14 |

Table S5. Gaussian distribution results of Young's modulus obtained from nanoindentation on shale with increasing maturity (Easy*R*_o_%).

| Easy*R*_o_（%） | Phase 1 | | | Phase 2 | | | Phase 3 | | |
| --- | --- | --- | --- | --- | --- | --- | --- | --- | --- |
|  | *E* (GPa) | *H* (GPa) | *f*  (%) | *E* (GPa) | *H* (GPa) | *f*  (%) | *E* (GPa) | *H* (GPa) | *f*  (%) |
| 0.55 | 19.3 | 0.27 | 70.8 | 26.6 | 0.57 | 23.1 | 48.9 | 5.44 | 6.1 |
| 0.66 | 23.1 | 0.44 | 75.6 | 33.9 | 1.53 | 23.2 | 110.5 | 16.5 | 1.1 |
| 0.75 | 22.6 | 0.38 | 79.5 | 34.4 | 2.44 | 18.6 | 139.6 | 16.4 | 1.8 |
| 0.87 | 21.6 | 0.35 | 50.5 | 31.8 | 0.7 | 39.6 | 34.6 | 1.85 | 9.9 |
| 1.04 | 24.7 | 0.51 | 84.8 | 43.9 | 2.34 | 11.5 | 83.3 | 11.6 | 3.7 |
| 1.26 | 24.7 | 0.39 | 62.0 | 31.0 | 0.81 | 30.2 | 47.4 | 3.07 | 7.8 |
| 1.52 | 25.0 | 0.71 | 78.3 | 36.9 | 2.33 | 18.3 | 75.4 | 12.0 | 3.4 |
| 1.82 | 25.1 | 0.8 | 81.7 | 45.1 | 4.87 | 17.4 | 118.1 | 13.9 | 0.9 |
| 2.16 | 26.6 | 0.66 | 70.7 | 38.5 | 2.06 | 25.4 | 81.2 | 9.91 | 4.0 |
| 2.91 | 29.8 | 0.86 | 54.3 | 37.8 | 1.63 | 33.0 | 58.7 | 4.83 | 12.7 |
